# Genetic monitoring and complex population dynamics: insights from a 12-year study of the Rio Grande silvery minnow

**DOI:** 10.1111/j.1752-4571.2011.00235.x

**Published:** 2012-01-12

**Authors:** Megan J Osborne, Evan W Carson, Thomas F Turner

**Affiliations:** Department of Biology and Museum of Southwestern Biology, University of New MexicoAlbuquerque, New Mexico, USA

**Keywords:** conservation genetics, genetic monitoring, population genetics – empirical

## Abstract

The endangered Rio Grande silvery minnow persists as a remnant population in a highly fragmented and regulated arid-land river system. The species is subject to dramatic annual fluctuations in density. Since 2003, the wild population has been supplemented by hatchery-reared fish. We report on a 12-year (1999–2010) monitoring study of genetic diversity and effective population size (*N*_e_) of wild and hatchery stocks. Our goals were to evaluate how genetic metrics responded to changes in wild fish density and whether they corresponded to the number and levels of diversity of hatchery-reared repatriates. Genetic diversity and all measures of *N*_e_ in the wild population did not correlate with wild fish density until hatchery supplementation began in earnest. Estimates of variance and inbreeding effective size were not correlated. Our results suggest source–sink dynamics where captive stocks form a genetically diverse source and the wild population behaves as a sink. Nevertheless, overall genetic diversity of silvery minnow has been maintained over the last decade, and we attribute this to a well-designed and executed propagation management plan. When multiple factors like environmental fluctuation and hatchery supplementation act simultaneously on a population, interpretation of genetic monitoring data may be equally complex and require considerable ecological data.

## Introduction

Demographic monitoring is used to evaluate conservation status, record changes in abundance, and assess outcomes of various management actions that affect species vulnerable to extinction or exploitation. Demographic monitoring can entail labor-intensive and costly direct estimation of key population parameters, such as recruitment, survival, and population size and their trajectories through time. The advent of sensitive and inexpensive genetic methods has prompted several authors to propose genetic monitoring as a lower-cost alternative (or complement) to demographic monitoring of key population parameters that assess conservation status, including abundance (Lavery and Keenan 1995; Ovenden et al. 2007; Schwartz et al. 2007; Long et al. 2008; Palstra and Ruzzante 2008). Genetic monitoring refers to the estimation of population genetic parameters, such as gene diversity, heterozygosity, allelic richness, and genetic effective size (*N*_e_), across a contemporary time series (Schwartz et al. 2007). These metrics are important indicators of the long-term adaptive potential and extinction vulnerability of imperiled species.

The rationale for genetic monitoring as a valuable management tool is that genetically based metrics provide scientifically robust insights into processes that affect standing levels of genetic diversity, offer defined benchmarks for assessing risks to a species persistence in the short- and long term, and, in principle, are linked explicitly to demographic changes in the population. For example, the relationship between genetic effective size, *N*_e_, and census size, *N*_c_, has important implications for conservation and management and has thus received considerable attention (Luikart et al. 2010). *N*_e_ can be defined as the size of an ideal population (Fisher 1930; Wright 1931) that experiences the same rate of change of allele frequencies (i.e., genetic drift) (*N*_eV_) or heterozygosity (*N*_eI_) over time as the real population (Crow and Kimura 1970). The most commonly used *N*_e_ estimates are inbreeding (*N*_eI_) and variance (*N*_eV_) effective population size. These measures of *N*_e_ track different facets of genetic change and do not estimate effective size in exactly the same generation (Waples 2005).

Many, if not most, papers that have evaluated the efficacy of genetic monitoring as a conservation and management tool have been theoretical or simulation studies (e.g., Antao et al. 2010; Waples and Do 2010) rather than ones based on empirical data (but see Ovenden et al. 2007). A recent simulation study (Antao et al. 2010) suggested that both single-sample and temporal-method estimators of *N*_e_ can reliably detect moderate to severe population declines. Furthermore, the relatively few empirical studies conducted to date have generally focused on single events (such as genetic bottlenecks) that may impinge on abundance and genetic diversity of the target species and considered relatively few time points (e.g., Ovenden et al. 2007; Chevolot et al. 2008; Karaiskou et al. 2011 but see Fraser et al. 2007; Eldridge and Killebrew 2008). In wild populations, especially those that are actively managed, there are often multiple factors that simultaneously act on a population, sometimes in ways that confound simple interpretation, especially if temporal samples are separated by many years. For example, many threatened, endangered, and exploited fish species experience dramatic population fluctuations and variation in recruitment from year to year, whilst at the same time their census numbers are bolstered by supportive breeding. Supportive breeding programs involve breeding and/or rearing individuals in captivity until they reach a less vulnerable life stage and size, at which time individuals are released to increase the census size of vulnerable populations.

In this paper, we report on results from 12 consecutive years of genetic monitoring conducted on the endangered Rio Grande silvery minnow (*Hybognathus amarus*). Historically, the species was distributed widely in the Rio Grande from northern New Mexico to the Gulf of Mexico, and in the Pecos River from above Sumner Reservoir (New Mexico) to the confluence of the Rio Grande in Texas (Pflieger 1980). It was extirpated from the Pecos River in the late 1960s, and the last collection was made from the lower Rio Grande, Texas, in the late 1950s (Bestgen and Propst 1996). The remnant population is restricted to a ∼280-km stretch of the Rio Grande from downstream of Cochiti Dam to Elephant Butte Reservoir, New Mexico. This stretch of river is bisected by three water diversion structures that define distinct river reaches (from north to south: Angostura, Isleta, and San Acacia). The current range is <5% of the historical range of the species. Rio Grande silvery minnow was listed under the Endangered Species Act in 1994 (U.S. Department of Interior 1994) and, according to demographic surveys, has since experienced several orders of magnitude fluctuations in density over the past two decades (U.S. Fish and Wildlife Service 2010). The wild population of Rio Grande silvery minnow is now heavily managed, with an extensive supportive and captive breeding program that has been in operation for almost a decade. Through this program, in excess of 1.1 million fish have been released throughout the current range of the species in New Mexico. Additionally, the species has been recently reintroduced to the Big Bend region in Texas where it occurred historically (Bestgen and Propst 1996).

Annual genetic monitoring of Rio Grande silvery minnow began in 1999. Roughly 5000 fish have been genotyped (at nine microsatellite loci and the mtDNA-ND4 gene) from throughout the remaining native range of the species, in addition to genotyping of repatriated individuals collected since the inception of the supportive breeding and supplementation program in 2002. Using these data and existing knowledge of life history and recent population trends in Rio Grande silvery minnow, we tested several simple predictions based on premises underpinning genetic monitoring. In regard to life history, the species is characterized by a type III survivorship curve, a 1:1 sex ratio, and a generation time that is roughly 1 year (Turner et al. 2006 and references therein).

Previously, we have shown that the wild population of Rio Grande silvery minnow has a low variance effective to census size ratio (Alò and Turner 2005), and we have hypothesized that this is because of the interaction of pelagic early life stage and river fragmentation (Osborne et al. 2005; Turner et al. 2006). Briefly, buoyant eggs and larvae are passively transported downstream (by river currents) from spatially distinct spawning sites, where they either pass through diversion dams or are retained in the natal reach to recruit to the parental stock. Adults fishes aggregate prior to spawning, and parents within an aggregation comprise a subset of the total genetic variation in a particular reach. Likewise, larvae retained in the natal reach themselves comprise a subset of the total genetic variation depending on the number of (and variance among) aggregates that retained larvae in that reach, plus the number of larvae that immigrated from upstream to recruit into the recipient reach. Because downstream transport rates and distances (>150 km) are likely to be large (Dudley and Platania 2007b), there is no appreciable genetic divergence that can be attributed to differences among river reaches (Osborne et al. 2005).

The silvery minnow appears to be genetically depauperate compared to several related and ecologically similar cyprinid fishes. For example, only 15 distinct mtDNA-haplotypes have been identified. By comparison, pelagic-spawning species such as Pecos bluntnose shiner (*Notropis simus pecosensis*) and Arkansas River shiner (*Notropis girardi*) were documented to contain 52 and 51 haplotypes among 1361 and 366 samples, respectively (Osborne and Turner 2009; Osborne et al. 2010a), in similar but unfragmented habitats in adjacent Pecos River.

In this study, we tested the following predictions across a 12-year time series of genetic data: (i) wild fish density, genetic diversity metrics, and all measures of effective size are positively correlated; (ii) supplementation provides a buffer against loss of diversity; hence, genetic variability has been maintained despite population declines in the wild; (iii) effects of population supplementation on genetic effective size depends on the source repatriated fishes (i.e., wild-caught eggs versus captive-bred); and (iv) captive fish broodstock composition (number of breeding pairs) and a point estimate of effective size, *N*_eD_, are positively correlated.

## Methods

### Sampling – Rio Grande population

Rio Grande silvery minnow were sampled from the Rio Grande annually from 1999 to 2010 (between December and April – adult fishes sampled prior to reproduction). In addition, 43 individuals obtained from the Museum of Southwestern Biology Division of Genomic Resources (studied previously in Cook et al. 1992 and referred to here as the 1987 sample) were genotyped. With the exception of the 1987 and 1999 collections, sample sizes are reflective of the species abundance in the wild (i.e., similar sampling effort was applied each year). Collections were made throughout the current distribution of Rio Grande silvery minnow that extends from Cochiti reservoir to Elephant Butte reservoir in New Mexico (Table 1) and included collections from multiple localities within each of three river reaches: Angostura (reach length = 65 kms), Isleta (reach length = 86 kms), and San Acacia (reach length = 92 kms). Rio Grande silvery minnow were collected by seining and occasional backpack electrofishing. Fishes were anesthetized with MS-222 (Tricaine methane sulfonate 200 mg/L river water) at the site of capture. A small piece of caudal fin was removed from each individual and preserved in 95% ethanol. Fishes were allowed to recover in untreated river water prior to release. Fin clips were also taken from 27 different captive stocks (seven stocks from captive-reared wild-caught eggs and 20 stocks from captive spawning) sampled between 2000 and 2008 (supplementation did not occur in 2009). We use the term ‘wild’ to refer to unmarked fish sampled directly from the Rio Grande. ‘Wild’ fish were hatched in the Rio Grande but could have had wild and/or captive-bred/reared parents. Fishes that originated from the hatchery were marked prior to release into the wild with an implanted elastomer tag that allowed visual distinction from wild fish in the field. Detailed information regarding the captive propagation and augmentation program is provided as Supporting Information (Appendix S1).

**Table 1:** Number of wild samples collected by year and river reach (Angostura, Isleta, and San Acacia). MSB Catalogue number indicates that voucher specimens are deposited at the Museum of Southwestern Biology, University of New Mexico

	MSB Cat.	Angostura	Isleta	San Acacia
1987	MSB4636, MSB*	15	–	28
1999	MSB49213	–	–	46
2000	MSB49216-19	–	–	194
2001	MSB49221	–	65	63
2002	–	67	121	201
2003	–	71	65	33
2004	–	141	15	6
2005	–	190	109	95
2006	–	95	143	145
2007	–	48	128	42
2008	–	165	191	123
2009	–	175	153	150
2010	–	149	146	151

MSB Catalogue number indicates that voucher specimens are deposited at the Museum of Southwestern Biology, University of New Mexico.

* The 1987 collection from the Angostura reach is uncatalogued.

### Molecular methods – microsatellites

Total nucleic acids, including genomic DNA and mitochondrial DNA (mtDNA), were extracted from air-dried fin clips, using proteinase-K digestion and phenol/chloroform extractions (Hillis et al. 1996). Individuals were genotyped at nine microsatellite loci: *Lco1*, *Lco3*, *Lco6*, *Lco7*, *Lco8* (Turner et al. 2004), *Ca6* and *Ca8* (Dimsoski et al. 2000) and *Ppro118* and *Ppro126* (Bessert and Ortí 2003). The following pairs of loci were amplified using multiplex PCR: *Lco1/ Ca6* and *Lco6*/*Lco7* [1× PCR buffer, 3 mm MgCl_2_, 125 μm deoxyribonucleotide triphosphates (dNTPs), 0.40–0.50 μm each primer, 0.375 units *Thermus aquaticus* (*TAQ*) polymerase], *Lco3* and *Lco8* (1× PCR buffer, 2 mm MgCl_2_, 125 μm dNTPs, 0.40–0.50 μm each primer, 0.375 units *TAQ*) and *Ppro 118*/*Ppro126* (1× PCR buffer, 3 mm MgCl_2_, 125 μm dNTPs, 0.40–0.50 μm each primer, 0.375 units *TAQ*). *Ca8* was amplified alone (1× PCR buffer, 3 mm MgCl_2_, 125 μm dNTPs, 0.50 μm each primer, 0.375 units *TAQ* polymerase). PCR cycling conditions for most loci were as follows: one denaturation cycle of 92°C for 2 mins followed by 30 cycles of 90°C for 20 s, 50°C for 20 s, 72°C for 30 s. For *Ppro 118*/*Ppro126*, cycling conditions were one denaturation cycle of 92°C for 2 mins followed by 30 cycles of 90°C for 20 s, 60°C for 20 s, 72°C for 30 s. *Ppro118* is a complex repeat microsatellite with alleles encompassing a broad size range. For this reason, a subset of samples that appeared homozygous at locus *Ppro118* were amplified again to verify allele designations and to minimize the likelihood of large allele dropout. Primer concentrations in multiplex reactions were adjusted to facilitate equal amplification of both loci. Prior to electrophoresis, 1 μL of PCR product was mixed with 10 μL of formamide and 0.3 μL of HD400 size standard and denatured at 93°C for 5 min prior to loading. PCR products were run on an ABI 3100 automated capillary sequencer. Genotype data were obtained using Genemapper version 4.0 (Applied Biosystems, Carlsbad, CA).

### MtDNA-ND4

A 295-base pair (bp) fragment of the mtDNA ND4 gene from each individual was amplified in a 10-μL reaction containing 1 μL template DNA, 1 μL 10× reaction buffer, 2 mm MgCl2, 125 μm dNTPs, 0.5 μm forward (5′-GAC CGT CTG CAA AAC CTT AA-3′) and reverse (5′-GGG GAT GAG AGT GGC TTC AA-3′) primers, and 0.375 U *TAQ*. PCR conditions were 90°C initial denaturation for 2 mins followed by 30 cycles of 90°C for 30 s, 50°C for 30 s, and 72°C for 30 s. Nucleotide sequence variation among individual fragments was visualized with single-strand conformational polymorphism analysis (Sunnucks et al. 2000), and representative haplotypes from each gel (∼20%) were verified by direct sequencing using an ABI 3100 DNA Sequencer (Genecodes, Ann Arbor, MI). Genbank Accession numbers are provided in Alò and Turner (2005) and Moyer et al. (2005). Three additional haplotypes were identified (Genbank Accession numbers: JN543958–JN543960).

### Statistical analysis

Microsatellite data were checked for errors, using Microsatellite Toolkit (add-in for Microsoft Excel, written by S. Park, available at http://animalgenomics.ucd.ie/sdepark/ms-toolkit/. The computer program Micro-Checker (Van Oosterhout et al. 2004) was used to examine data for scoring errors associated with stuttering, large allele dropout, and presence of null alleles. Genepop (Raymond and Rousset 1995) was used to assess whether there were significant departures from Hardy–Weinberg equilibrium (HWE) using the procedure of Guo and Thompson (1992). Global tests for linkage disequilibrium were conducted for all pairs of loci, using fstat vers. 2.9.3.1 (Goudet 1995). Sequential Bonferroni correction (Rice 1989) was applied to account for inflated type 1 error rates associated with multiple simultaneous tests. For each microsatellite locus and population inbreeding coefficients (*F*_IS_) were obtained using fstat. Estimates of unbiased gene diversity (*h*) were obtained using Arlequin vers. 3.11 (Excoffier et al. 2005) for mitochondrial DNA data. Haplotype richness (Petit et al. 1998) was obtained using the program Contrib vers. 1.02 (available at http://www.pierroton.inra.fr/genetics/labo/Software/Contrib/), which uses a rarefaction approach to correct for unequal sample sizes.

In some cases, sample sizes differed between collections particularly between some samples collected early in the study and those collected more recently. As the number of alleles and expected heterozygosity are dependent on sample size, we used resampling to examine the effect of sample size on diversity measures. For microsatellites, 1000 random subsamples (*n* = 43 in 1987) were drawn without replacement from each temporal sample. Diversity and 95% CIs were calculated for each locus across subsamples and a mean was obtained across loci for each statistic [corrected number of alleles (*N*_ac_), gene diversity (Nei 1987) (*H*_ec_), heterozygosity (*H*_oc_)]. This analysis was conducted in the R statistical package (http://www.r-project.org; R script available on request). Standard diversity measures (*H*_e_, *H*_o_, and *A*_R_) for microsatellites are provided as Supporting information (Table S2). To facilitate comparisons among collections obtained from different river reaches across years, we repeated the resampling procedure for microsatellite data in R where diversity measures were based on *n* = 15 (2004 Isleta) and the smallest sample *n* = 6 (2004 San Acacia) was excluded. Corrected measures of diversity were compared between river reaches using a nonparametric Kruskal**–**Wallis one-way anova on ranks implemented in SigmaPlot vers. 11.0 (Systat Software Inc., San Jose, CA).

### *F*-statistics

Weir and Cockerham’s (1984)*F*-statistics (microsatellites) and Φ-statistics (mtDNA) were calculated in Arlequin vers. 3.11 (Excoffier et al. 2005). Hierarchical analysis of molecular variance (amova) was conducted to test whether a significant proportion of genetic variance was partitioned into components attributable to differences among wild, captive-spawned, and captive-reared stocks (i.e., wild-caught eggs were the source) (*F*_CT_, *Φ*_CT_), among samples within these three groups (*F*_SC_, *Φ*_SC_) and among all samples (*F*_ST_, *Φ*_ST_). *P*-values for all statistics were generated using bootstrapping (1000 permutations), as implemented in Arlequin.

### Estimation of genetic effective size

Variance genetic effective size (*N*_eV_) and 95% CIs were estimated from temporal changes in microsatellite allele frequencies across annual samples, using the temporal method (Nei and Tajima 1981; Waples 1989) implemented in NeEstimator (Peel et al. 2004) and a pseudo-maximum-likelihood procedure implemented in mlne version 2.3 (Wang 2001). Estimates of *N*_eV_ were corrected for sample size variation by subtracting the expected variance attributed to sampling from the observed temporal variance in allele frequencies (Waples 1989; eq. 12). Highly polymorphic loci with many rare alleles, as is typical of microsatellites, can be subject to biased estimates of variance effective size, *N*_eV_ (Hedrick 1999; Turner et al. 2001). To account for this potential bias, the unbiased estimator, *F*_S_ (Jorde and Ryman 2007), as implemented in TempoFs (http://www.zoologi.su.se/_ryman), was used to estimate *N*_eV_. Rio Grande silvery minnows were sampled under Plan I (prior to reproduction, with replacement) for all methods; therefore, calculations of *N*_eV_ via TempoFs required an estimate of census size (*N*_c_). No reliable, long-term data (i.e., spanning the entire sampling period) were available for *N*_c_, so each pairwise comparison in TempoFs was run under the following two *N*_c_ scenarios: a ‘crashed’ (*N*_c_ = 10 000) and a ‘large’ (1 000 000 individuals) population. The former value is lower than any census size estimate to date, and the latter is within the order of magnitude for which larger *N*_c_ have been recorded (Dudley et al. 2011). In all comparisons, differences in mean *N*_eV_ were negligible between the *N*_c_ = 10 000 and *N*_c_ = 1 000 000 scenarios, but lower and upper confidence intervals were slightly larger for the latter. Only the most conservative *N*_eV_ estimates (i.e., based on *N*_c_ = 1 000 000) are reported herein; *N*_eV_ and confidence intervals calculated under both *N*_c_ = 10 000 and 1 000 000 can be obtained by request. Jackknife estimation over all loci was used to calculate *N*_eV_ and associated 95% CI.

For all methods, we assumed that migration from outside the study area did not affect estimates of *N*_e_. We equated the number of years separating a pair of samples with the number of generations elapsed between samples because Rio Grande silvery minnow have essentially nonoverlapping generations (based on unpublished population monitoring data of R. K. Dudley and S. P. Platania). However, to account for small but known deviations from the discrete generation model (*G* = 1.27), we corrected consecutive estimates of *N*_e_ and *N*_ef_ for overlapping generations (Turner et al. 2006; Osborne et al. 2010b), using the analytical method of Jorde and Ryman (1995, 1996). In addition to consecutive pairwise estimates, we also present comparisons between the 1987 and 1999 samples to provide historical context for the contemporary estimates. As these samples (1987–1999) were collected more than 3–5 generations apart, the drift signal should be sufficiently large relative to sampling biases associated with age-structure such that correction for overlapping generations is unnecessary (Waples and Yokota 2007).

In addition to the estimates of *N*_eV_, we used the linkage disequilibrium method (Hill 1981) to estimate *N*_eD_ from microsatellite DNA data for each annual sample (including wild, captive-spawned, and wild-caught eggs), using the program ldne (Waples and Do 2008) and methods described in Osborne et al. (2010b). Single-sample *N*_e_ methods (such as those provided by ldne) yield an estimate of the effective number of parents that produced the progeny from which the sample is drawn, and most closely approximate inbreeding effective size, *N*_eI_ (Laurie-Ahlberg and Weir 1979; Waples 2005).

For mtDNA data, variance effective size for the female portion of the population (*N*_ef_) was estimated with temporal (Turner et al. 2001) and pseudo-maximum-likelihood (mlne) methods. TempoFs was not used for mtDNA data because it assumes diploidy (Jorde and Ryman 2007).

### Effects of demography, environment, and supplementation

**Figure 1:**
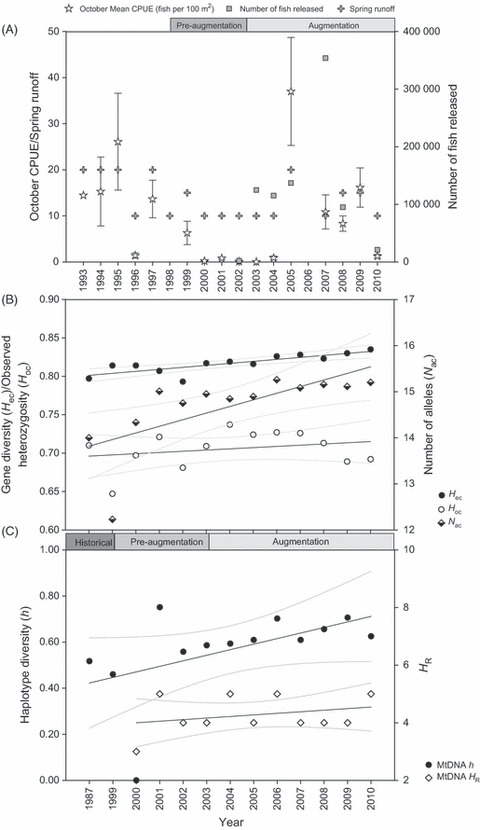
(A) Wild Rio Grande silvery minnow October density (catch per unit effort: fish per 100 m^2^), number of fish released for population supplementation and spring runoff (arbitrary values of 10–20). (B) Microsatellite DNA diversity metrics obtained using resampling: expected heterozygosity (*H*_ec_), observed heterozygosity (*H*_oc_), and mean number of alleles (*N*_ac_). (C) Mitochondrial DNA diversity metrics: haplotype diversity (*h*) and haplotype richness (*H*_R_). Linear regressions are shown with associated 95% CIs.

To enable comparisons between metrics obtained from genetic data, environmental conditions, demography, and supportive breeding and supplementation, we plotted three parameters: (i) fall recruitment [given by mean October catch per unit effort (CPUE) provided by American Southwest Ichthyological Researchers], (ii) spring runoff, and (iii) number of fish stocked as part of the supportive breeding program for reference purposes (Fig. 1A). Spring runoff was treated as a categorical variable and ranged from 10 to 20, where an arbitrary value of 10 was given if spring flows were <3000 cubic feet per second (cfs) for more than 14 days, 15 when flows were more than 3000 cfs for more than 14 days but <30 days, and a value of 20 was given if flows were >3000 cfs for more than 30 days at the United States Geological Survey (USGS) Albuquerque stream gauge (08330000) during the months of May and June. Spring runoff is an important environmental metric because increases in flow are a spawning cue for Rio Grande silvery minnow (Platania and Altenbach 1998; Platania and Dudley 2006) and there is a strong positive correlation between peak discharge and duration of high flows during the spawning season (May and June) and mean October densities (U.S. Fish and Wildlife Service 2010 and references therein). Numbers of fish repatriated from protective custody to the wild population were provided by US FWS (J. Remshardt, personal communication).

To test the first prediction (density, genetic diversity, and genetic effective size are positively correlated), CPUE data were log-transformed according to Sokal and Rohlf (1997), following Dudley and Platania (2007a), and linear regressions were then used to examine the presence and strength of relationships among these key parameters. Spring runoff data are strongly correlated with October CPUE data (Dudley and Platania 2007a); thus, the former was not used in regressions. CPUE and *N*_e_ estimates were paired for the generation that was affected by the conditions, such that CPUE and runoff in year *t* were matched with *N*_e_ estimates in year *t −* 1. For example, a temporal estimate calculated from adult samples collected prior to breeding in 2000 (gen *t*_0_) and 2001 (gen *t*) provides an estimate of effective size in generation *t*_0_ (i.e., 2000); for a detailed explanation, see Waples (2005). Hence, the runoff and fall recruitment data of 1999 would be paired with this estimate. Single-sample (ldne) and temporal measures of effective size do not refer to exactly the same generation (Waples 2005). Specifically, the ldne estimate for the 2000 sample refers to *N*_eI_ for the 1999 generation; thus, estimates would be paired with relevant spring runoff and fall recruitment data for the year prior to this (i.e., 1998).

To test the second prediction (population supplementation acts as a buffer against loss of diversity), sample-size-corrected measures of diversity were compared between years prior to population supplementation (1999–2003) and years after supplementation commenced (2004–2010) by Mann–Whitney *U*-tests (SigmaPlot vers. 11.0; Systat Software Inc.). Likewise, corrected diversity statistics for the San Acacia reach were also compared before and after the commencement of supplementation. This analysis was not performed for the Isleta or Angostura reaches because of the small number or absence of samples for the presupplementation years.

To examine the third prediction [effects of population supplementation are dependent on broodstock source (wild-caught eggs or captive-spawned)], we compared diversity metrics (obtained using resampling for microsatellites) among fishes reared from wild-caught eggs to those produced with captive spawning using a Mann–Whitney *U*-test. Estimates of *N*_eD_ were also compared among these samples. Data were examined qualitatively for perceptible changes in genetic effective size that could be attributed to supplementation strategy (i.e., with either wild-caught eggs or captive-spawned fish).

The final prediction that captive fish broodstock composition and point estimates of effective size (*N*_eD_) will be positively associated was tested using ordinary least-squares linear regression of broodstock effective size (calculated using the equation *N*_e_ = *4N*_m_*N*_f_*/*[*N*_m_ *+ N*_f_] to account for unequal sex ratio) and estimates of *N*_eD_.

## Results

### Microsatellites – genetic diversity

**Figure 2:**
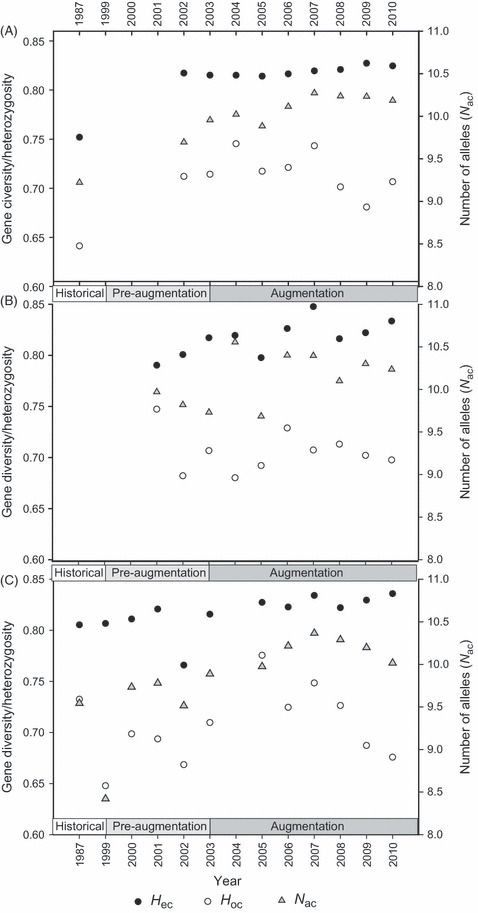
Diversity metrics from microsatellite data obtained using resampling (*H*_ec_, *H*_oc_, and *N*_ac_) by river reach (A) Angostura, (B) Isleta, and (C) San Acacia are provided.

A total of 5056 fish were genotyped for nine microsatellite loci over the 12-year study. Microsatellite locus *Ca6* was the least variable with 10 alleles detected across all populations, whereas *Ppro118* was the most variable with 63 alleles. After sequential Bonferroni correction for multiple comparisons, there were 127 departures from HWE among 360 comparisons. Fifty-one of these involved wild samples, 20 involved fish reared from wild-caught eggs, and 56 involved captive-spawned stocks. Locus *Lco8* accounted for 36 departures from HWE, which was the highest number of significant tests for any locus. Four loci (*Lco3*, *Lco6*, *Ca6*, *Ppro126*) conformed to HWE in all or nearly all comparisons. Micro-Checker suggested that null alleles probably caused departures from HWE. We adjusted allele frequencies in Microchecker to account for null alleles and reran a subset of analyses and did not obtain different results (see Turner et al. 2006). We therefore conclude that the presence of null alleles does not appreciably affect our estimates of diversity or *N*_e_. Across all samples, there was no evidence of linkage disequilibrium among loci after Bonferroni correction. Observed gene diversity, heterozygosity, and allelic richness calculated for temporal samples and the values corrected to the smallest sample size (Fig. 1B) were strongly correlated (gene diversity and heterozygosity ρ_2_ = 1, *P* < 0.00001; allelic diversity ρ_2_ = 0.827, *P* < 0.00001). Metrics of genetic diversity did not differ significantly by river reach (*H*_ec_: *P* = 0.740, *H*_oc_: *P* = 0.869, *N*_ac_: *P* = 0.327) (Fig. 2A–C). In all reaches, there was a substantial decrease in *N*_ac_ in 2005 compared to other years.

### Mt-DNA – genetic diversity

**Figure 3:**
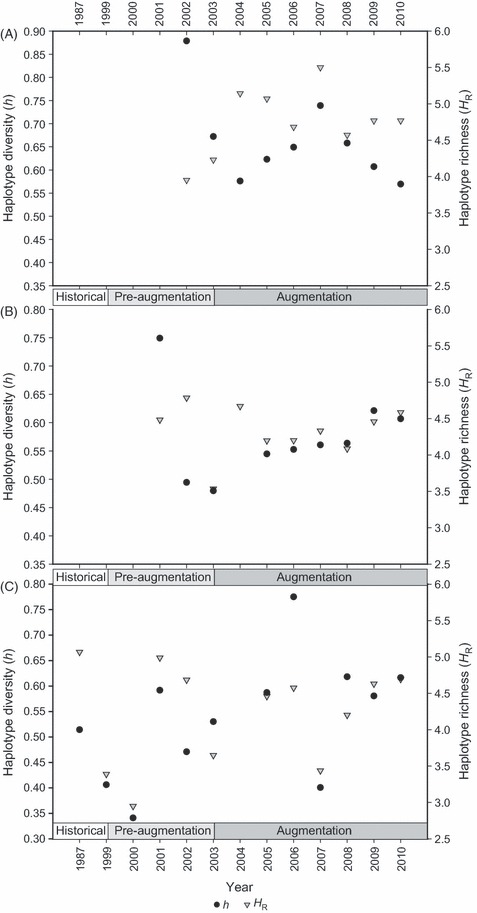
Haplotype diversity (*h*), haplotype richness (*H*_R_), metrics from mitochondrial DNA-ND4 by river reach (A) Angostura, (B) Isleta, and (C) San Acacia.

A total of 15 mtDNA haplotypes were identified among 4915 individuals assayed. Nucleotide sequence divergence among haplotypes was low, with one to six substitutions among them. Haplotype A was the most common in all samples except Cs-An-02 (captive-spawned) which was monomorphic for haplotype D (Table 2). Three haplotypes (C, D, F) were present at moderate frequencies (>5%), and 11 haplotypes were considered rare (present at frequencies <5%). Gene diversity (*h*_c_) was highest in the 1987 samples (*h*_c_ = 0.743) and lowest in the 2000 sample (*h* = 0.364) (Fig. 1C, Table 3). Haplotype diversity (*P* = 0.033) differed significantly by river reach whilst *H*_R_ did not (*P* = 0.066) (Fig. 3).

**Table 2:** Mt-DNA haplotype frequencies across all wild and captive (wild-caught eggs and captive-spawned) stocks

	Mt-DNA-ND4 haplotypes														
				
	A	C	D	E	F	K	I	J	M	N	P	O	Q	S	T
Wild															
1987	0.459	0.162	0.162	0.054	0.081	0.027	–	–	0.054	–	–	–	–	–	–
1999	0.750	–	0.114	0.068	0.045	0.023	–	–	–	–	–	–	–	–	–
2000	0.790	0.008	0.048	0.048	0.097	0.008	–	–	–	–	–	–	–	–	–
2001	0.607	0.090	0.057	0.033	0.098	0.074	0.008	0.016	0.008	–	–	0.008	–	–	–
2002	0.556	0.199	0.137	0.010	0.059	0.034	–	0.003	–	–	–	0.003	–	–	–
2003	0.671	0.054	0.150	0.030	0.054	0.012	–	0.006	0.006	–	–	0.018	–	–	–
2004	0.596	0.087	0.106	0.019	0.075	0.050	0.012	–	0.019	–	0.006	0.031	–	–	–
2005	0.598	0.126	0.088	0.028	0.086	0.018	0.015	0.003	0.028	–	–	0.010	–	–	–
2006	0.587	0.135	0.093	0.048	0.048	0.048	0.003	–	0.029	–	–	0.008	–	–	0.003
2007	0.628	0.110	0.083	0.023	0.087	0.037	0.005	–	0.005	–	–	0.018	0.005	–	–
2008	0.635	0.120	0.079	0.026	0.067	0.045	0.004	–	0.009	–	0.002	0.006	–	0.006	–
2009	0.614	0.140	0.076	0.028	0.064	0.034	0.006	0.004	0.019	–	0.002	0.011	–	0.002	–
2010	0.562	0.124	0.097	0.032	0.069	0.053	0.014	–	0.016	–	–	0.032	–	–	–
Wild-caught eggs															
WcE-01	0.573	0.197	0.051	0.064	0.064	0.032	–	–	0.013	0.006	–	–	–	–	–
WcE-SA-01	0.569	0.137	0.059	0.059	0.098	0.078	–	–	–	–	–	–	–	–	–
WcE-An-02	0.653	0.020	0.327	–	–	–	–	–	–	–	–	–	–	–	–
WcE-SA02	0.488	0.225	0.050	0.013	0.138	0.050	–	–	0.038	–	–	–	–	–	–
WcE-SA-03	0.490	0.078	0.196	0.059	0.098	0.039	–	–	0.020	–	–	0.020	–	–	–
MJO07-005	0.604	0.094	0.019	0.019	0.170	0.075	–	0.019	–	–	–	–	–	–	–
MJO07-006	0.604	0.083	0.125	0.021	0.083	0.042	–	–	–	–	–	0.042	–	–	–
Captive-spawned															
MJO06-29	0.680	0.140	0.080	–	0.060	–	–	–	0.040	–	–	–	–	–	–
Cs-01	0.724	0.052	–	0.034	0.069	0.121	–	–	–	–	–	–	–	–	–
Cs-An-02	–	–	1.000	–	–	–	–	–	–	–	–	–	–	–	–
Cs-SA-02	0.434	0.075	0.170	0.132	0.170	–	–	–	–	–	0.019	–	–	–	–
Cs-04	0.596	0.255	0.021	–	0.043	0.064	–	–	–	–	0.000	0.021	–	–	–
TFT039	0.596	0.269	0.038	–	0.000	0.096	–	–	–	–	0.000	–	–	–	–
TFT04-23	0.617	0.043	0.191	–	0.000	0.043	–	–	–	–	0.000	0.106	–	–	–
TFT04-24	0.583	0.125	0.208	–	0.021	0.063	–	–	–	–	0.000	–	–	–	–
TFT04-25	0.434	0.057	0.113	0.057	0.283	0.057	–	–	–	–	0.000	–	–	–	–
TFT04-29	0.566	0.245	–	0.075	–	0.094	–	–	0.019	–	0.000	–	–	–	–
TFT04-30	0.400	0.333	–	–	–	0.244	–	–	–	–	0.022	–	–	–	–
TFT04-31	0.420	0.340	0.020	–	0.060	0.040	–	–	0.100	–	–	0.020	–	–	–
TFT05-06	0.500	0.360	0.020	–	0.020	0.080	–	–	0.020	–	–	–	–	–	–
TFT05-07	0.625	0.292	0.021	0.063	–	0.000	–	–	–	–	–	–	–	–	–
TFT05-08	0.592	0.082	–	0.102	–	0.224	–	–	–	–	–	–	–	–	–
TFT05-09	0.680	0.160	–	–	–	0.120	–	–	0.040	–	–	–	–	–	–
TFT05-11	0.623	0.057	0.113	0.019	0.170	–	–	–	0.019	–	–	–	–	–	–
MJO06-25	0.551	0.245	0.061	–	0.061	0.082	–	–	–	–	–	–	–	–	–
MJO06-28	0.400	0.140	0.220	–	0.220	0.020	–	–	–	–	–	–	–	–	–
MJO07-007	0.560	0.020	0.120	0.280	0.020	–	–	–	–	–	–	–	–	–	–

**Table 3:** Summary statistics obtained using a resampling approach are provided for microsatellite loci for wild, hatchery-reared wild-caught eggs (WcE), captively spawned (Cs) Rio Grande silvery minnow. Haplotype diversity (*h*) and haplotype richness (*H*_R_) are provided for mtDNA-ND4. Estimates of inbreeding effective size (*N*_eD_) and associated confidence intervals are also included

	Microsatellites								Mt-DNA			
					
Population	*N*	*H*_ec_	*H*_oc_	*N*_ac_	*F*_IS_	*N*_eD_	−95%	+95%	*N*	*h*	*H*_R_	No. Haps
Wild												
1987	43	0.797	0.710	14.000	0.111	∞	139.3	∞	37	0.743	6.0000	7
1999	46	0.814	0.647	12.229	0.210	∞	∞	∞	44	0.427	3.8160	5
2000	194	0.814	0.697	14.332	0.145	∞	∞	∞	124	0.364	3.3590	6
2001	128	0.807	0.721	15.008	0.107	2007.7	495.1	∞	122	0.609	6.0630	10
2002	389	0.793	0.681	14.752	0.143	1950.6	701.7	∞	387	0.630	4.1630	8
2003	169	0.817	0.709	14.951	0.134	2997.7	563.8	∞	167	0.524	4.8900	9
2004	162	0.819	0.737	14.845	0.100	595.5	357.2	1558.7	161	0.620	6.2770	10
2005	394	0.816	0.724	14.895	0.113	2724.3	1013.5	∞	396	0.610	5.6330	10
2006	383	0.826	0.727	15.259	0.122	2561.7	1291.4	34063.9	378	0.622	5.6700	10
2007	218	0.828	0.726	15.084	0.123	∞	1210.7	∞	218	0.579	5.3630	10
2008	474	0.823	0.713	15.156	0.135	4458.5	1478.5	∞	466	0.569	5.3010	11
2009	476	0.830	0.689	15.113	0.172	3607.6	1676.9	∞	472	0.592	5.6490	12
2010	440	0.835	0.692	15.202	0.172	∞	2022.8	∞	433	0.649	6.0870	9
Wild-caught eggs												
WcE-01*	178	0.818	0.651	14.803	0.206	1379.6	655.6	∞	157	0.627	6.999	8
WcE-SA-01	50	0.830	0.727	13.949	0.126	∞	238.3	∞	51	0.624	6.000	6
WcE-An-02	50	0.784	0.730	12.120	0.070	85.6	54.1	173.4	49	0.481	2.949	3
WcE-SA-02	81	0.818	0.680	14.947	0.171	∞	461.7	∞	80	0.702	7.376	8
WcE-SA-03	51	0.830	0.695	14.982	0.164	5008.5	307.6	∞	51	0.714	7.848	8
MJO-07-005	54	0.827	0.739	15.329	0.108	1065.0	195.9	∞	53	0.602	6.733	7
MJO-07-006	49	0.814	0.722	15.631	0.114	∞	520.6	∞	48	0.581	5.962	6
Captive-spawned												
MJO-06-29	50	0.803	0.745	11.368	0.074	42.2	28.7	68.7	50	0.517	5.000	5
Cs-01	64	0.794	0.658	12.807	0.172	43.7	35.6	55	58	0.460	4.982	5
Cs-An-02	51	0.685	0.675	8.463	0.015	21.6	14.9	32.5	51	0.000	1.000	1
Cs-SA-02	53	0.802	0.674	13.154	0.163	72.7	52.5	110.9	53	0.751	5.919	6
TFT039	51	0.806	0.700	12.766	0.133	106.3	56	433.5	52	0.558	3.995	4
Cs-04	50	0.824	0.691	14.082	0.163	65.5	45.7	105.7	47	0.586	5.911	6
TFT-04-23	50	0.779	0.683	11.641	0.124	20.4	16.5	25.4	47	0.593	4.996	5
TFT-04-24	48	0.828	0.717	11.749	0.135	40.2	29.7	57.8	48	0.609	4.949	5
TFT-04-25	50	0.810	0.768	11.643	0.053	24.9	20	31.5	53	0.702	5.934	6
TFT-04-29	54	0.839	0.762	14.024	0.092	∞	532.2	∞	53	0.609	4.903	5
TFT-04-30	56	0.826	0.726	14.689	0.121	323.1	134	∞	45	0.656	4.790	5
TFT-04-31	50	0.805	0.700	12.798	0.13	83.2	54.7	154.7	50	0.706	6.865	7
TFT-05-006	50	0.792	0.649	10.303	0.183	49.4	38.8	65.7	50	0.625	5.803	6
TFT-05-007	49	0.797	0.705	12.157	0.117	86.6	53.2	191.3	48	0.550	4.884	5
TFT-05-008	50	0.804	0.662	11.152	0.178	32.2	26.7	39.5	49	0.611	4.934	5
TFT-05-009	50	0.804	0.717	12.911	0.109	219.9	98.8	∞	50	0.506	3.996	4
TFT-05-011	51	0.808	0.693	12.543	0.144	136.6	81	354	53	0.573	5.853	6
MJO-06-25	50	0.813	0.721	14.853	0.115	184.5	110.1	487.9	49	0.635	4.934	5
MJO-06-028	50	0.805	0.705	12.395	0.125	87.6	57.2	164.3	50	0.738	4.996	5
MJO-07-007	50	0.813	0.739	13.156	0.091	60.4	48.3	78.5	50	0.605	4.869	5

Sample size (*N*) and average weighted inbreeding coefficient (*F*_IS_) and diversity statistics obtained using resampling approach: gene diversity (*H*_ec_), observed heterozygosity (*H*_oc_), and allelic (*N*_ac_) for microsatellites. *N*_eD_ estimates (based on nine microsatellite loci) and associated 95% confidence intervals (obtained using jackknifing) are given. For ND4 sample size (*N*), gene diversity (*h*), haplotype richness (*H*_R_), and observed number of haplotypes are given.

* WcE-01 sample was also collected from San Acacia but reared at Dexter National Fish Hatchery and Technology Center (WcE-SA-01 was reared at the Albuquerque Biopark). (An, Angostura; SA, San Acacia, numerals following refer to the years eggs were collected, for example WcE-SA-01 were wild-caught eggs collected from the San Acacia reach in 2001).

### *F*-statistics

#### Microsatellites

A small but significant portion of variance was explained by differences among wild, captive-spawned, and captive-reared samples (*F*_CT_ = 0.00052, *P* = 0.007). Samples within each of these groups also differed significantly from one another (*F*_SC_ = 0.0067, *P* < 0.00001), and a significant portion of variance was also explained by differences among samples irrespective of group affinity (*F*_ST_ = 0.0076, *P* < 0.0001).

#### MtDNA

A significant portion of variance was explained by differences among samples within groups (wild, captive-spawned, captive-reared) and among samples irrespective of groups (*Φ*_SC_ = 0.0558, *P* < 0.0001; *Φ*_ST_ = 0.0052, *P* < 0.0001) but not among groups (*Φ*_CT_ = −0.0037, *P* = 0.1359).

### Genetic effective size

**Figure 4:**
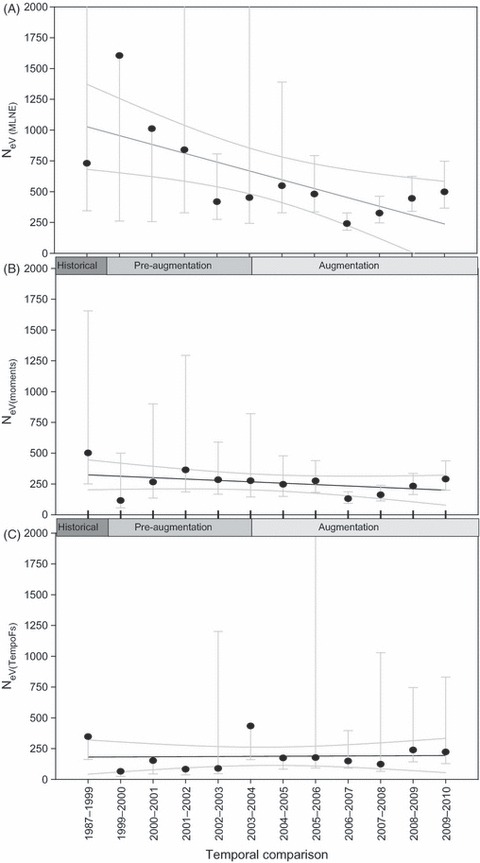
Variance effective size calculated from microsatellite DNA data using (A) mlne, (B) Moments-based, and (C) TempoFs methods and associated 95% CIs (absence of +95% error bars indicates upper bounds of infinity). Linear regressions are shown with associated 95% CIs.

Multi-locus temporal estimates of *N*_eV_ were calculated with all nine loci, and separately with *Lco8* removed because of concern that deviation from HWE at this locus could lead to spurious results. Estimates were essentially identical, so only those generated from the full data set are reported (estimates from the eight locus data set are available upon request). TempoFs indicated relatively low effective size (*N*_eV_ = 64–152) for pairwise, consecutive year comparisons between 1999 and 2003 (Fig. 4). Effective population size rebounded (*N*_eV_ = 433) in 2003–2004, but another reduction in *N*_e_ occurred from 2006 to 2008. The same pattern of fluctuation in *N*_e_ was detected with moments-based and mlne estimators. TempoFs estimates of *N*_eV_ were stable and above 200 from 2008 to 2010; similarly, moments and mlne estimates for this period were above 200 and 400, respectively. Overall, TempoFs estimates had wider upper confidence limits than moments and mlne estimates. Mean values of *N*_eV_ from the moments estimators were greater than from TempoFs but were still small for the 1999–2000 (*N*_eV_ = 115) and 2006–2007 (*N*_eV_ = 160) comparisons. In accordance with Antao et al. (2010), mlne estimates were higher than TempoFs and moments estimates.

**Figure 5:**
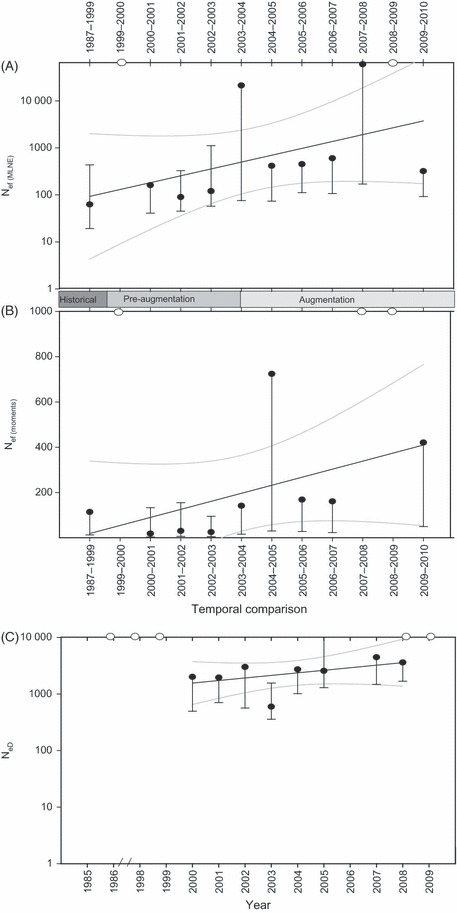
Variance effective size calculated from mitochondrial DNA-ND4 using (A) mlne (*y*-axis is a log scale), (B) moments-based methods and 95% CIs, and (C) estimates of *N*_eD_ and 95% CIs by year (*y*-axis is a log scale). Linear regressions are shown with associated 95% CIs. Estimates of infinity are indicated by open circles. Absence of +95% error bars indicates upper bounds of infinity.

Values of effective size based on linkage disequilibrium were generally larger in magnitude than those based on temporal-method estimators. For example, *N*_eD_ estimates of infinity were obtained for the 1987, 1999, 2000, 2007, and 2010 samples (Table 1, Fig. 5). In 2001 and 2002 (presupplementation), *N*_eD_ estimates were approximately 2000. In 2004, there was a marked decline in *N*_eD_ to 595 (95% CIs, 357–1559), with estimates rebounding in the years between 2005 and 2010 (2562-infinity). *N*_eD_ was also estimated without Lco8 (Table S3) but again, results did not differ appreciably from those obtained with all loci.

Female effective size based on temporal variance of mtDNA haplotype frequencies also fluctuated dramatically over the period of study. Between 2000 and 2003, *N*_ef_ was very low for moments-based (*N*_ef_ < 30) and mlne (*N*_ef_ < 100) (Fig. 5). Both estimators detected subsequent increases in *N*_ef,_ from 2004 to 2005, but the magnitude of increase was substantially different between them. The moments-based estimator detected a decline in *N*_ef_ for the 2005–2007 period whilst mlne showed a decline for pairwise estimates between 2004 and 2007. Both methods revealed large increases in *N*_ef_ for 2007–2009 periods and more recent reductions to 361 (mlne) and 565 (moments) for the 2009–2010 comparison.

## Testing predictions

**Figure 6:**
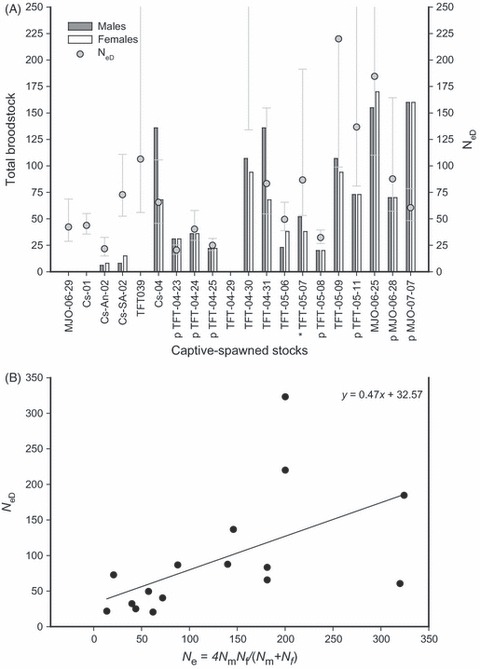
Estimates of *N*_eD_ calculated from microsatellite loci and broodstock information (A) Estimates of *N*_eD_ and number of male and female broodstock (B) Estimates of *N*_eD_ and *N*_e_ estimated by *N*_e_ = *4N*_m_*N*_f_*/(N*_m_*+N*_f_).

1. A positive relationship between density, diversity, and effective size is expected
  CPUE, diversity metrics (microsatellite and mtDNA), and *N*_e_ estimates calculated using temporal or linkage disequilibrium methods were not correlated over the entire time series. However, after supplementation began, *N*_eV_ moments estimates were positively correlated (Spearman’s rank order) with mlne estimates and TempoFs estimates (ρ_7_ = 0.76, *P* = 0.0384; ρ_7_ = 0.680, *P* = 0.0735, respectively), as expected. Furthermore, *N*_e_ estimates were not inter-correlated, except for a significant negative correlation between mlne and *N*_eD_ (ρ_9_ = −0.690, *P* = 0.047).
2. Supportive breeding and population supplementation buffers against loss of diversity
  Overall values of *H*_ec_ and *N*_ac_ were significantly greater (*P* = 0.005, 0.028) in wild samples collected after (2004–2010) than prior to (1999–2003) the commencement of the supplementation program as was *H*_R_ (*P* = 0.048). Two measures of diversity (*H*_ec_ and *N*_ac_) were significantly higher (*P* = 0.004 and 0.004, respectively) for fish collected from the San Acacia reach after commencement of supplementation, whilst *H*_oc_ was significantly less (*P* = 0.009) in these fish. There were no significant differences in haplotype diversity (*P* = 0.662) or haplotype richness (*P* = 0.126) in samples collected from the San Acacia reach before and after supplementation commenced.
3. The source of brood stock influences genetic effects of supplementation on the wild population.
  Diversity measures did not differ significantly between wild samples and samples derived from wild-caught eggs (*H*_ec_: *P* = 0.691; *H*_oc_: *P* = 0.663, *N*_ac_: *P* = 0.937; *h*: *P* = 0.383; *H*_R_: *P* = 0.052). *H*_ec_ and *N*_ac_ differed significantly between wild and captive-spawned (from hatchery broodstock) samples (*P* = 0.026 and <0.001, respectively) and between the captive-spawned and wild-caught eggs (*P* = 0.049 and 0.004, respectively), with wild and wild-caught eggs having greater genetic variation than captive-spawned samples. Haplotype richness was significantly lower in captive-spawned fish when compared with wild samples (*P* = 0.05) and wild-caught eggs (*P* = 0.009). Use of adults reared from wild-caught eggs for population supplementation was predicted to be associated with an increase in *N*_eV_. This was observed for the 2003–2004 TempoFs comparison. mlne and moments *N*_eV_ estimates did not change appreciably from the previous temporal comparison. Female *N*_eV_ (mlne) increased for the same time period, whilst the moments estimate increased for this comparison and the next temporal estimate.
4. Broodstock effective size is positively correlated with *N*_eD_
  As predicted, there was a positive linear relationship between the effective number of broodstock and estimates of *N*_eD_ (*r*^2^ = 0.308, *P* = 0.023, df = 15; Fig. 6). *N*_eD_ estimates for captive stocks raised from wild-caught eggs were typically larger than those obtained from captive spawning, with noninfinite estimates ranging from 86 to 5009 and 22 to 323, respectively. Of the 16 *N*_eD_ estimates for stocks produced from captive spawning, five estimates accurately reflected the known broodstock size. In eight instances, true broodstock size was within the 95% CIs of *N*_eD_ estimates, whilst nine estimates suggested smaller *N*_e_ than the number of breeders used (including paired matings) and two were overestimates. The slope from least-squares regression was ∼0.5, indicating that *N*_eD_ was approximately one-half of *N*_e_ explained by the number of brood fish and sex ratio variation among captive lots.

## Discussion

Identification of relationships between demography and genetic variation is a crucial but difficult task in conservation and management of exploited or endangered organisms, especially those subject to hatchery-based supplementation, and/or those that experience highly variable population sizes and fluctuating environmental conditions. Here, we evaluated the ability of genetic monitoring to recover such potentially complex interactions in a species that has been subject to all three of these effects in recent times. Our study used long-term, empirical data from genetic (1987, 1999–2010) and population monitoring (1993–2010) in an attempt to synthesize this information with temporal records of environmental conditions, captive breeding, and wild population supplementation. A notable increase in mean values of key diversity metrics and a coincident decrease in inter-annual variability in these metrics indicated that population supplementation has been the single most important factor that has influenced trajectories of genetic diversity over the last decade in Rio Grande silvery minnow. Large-scale repatriation of hatchery-reared fishes into the Rio Grande has potentially obscured (and ameliorated) most genetic effects of density fluctuations and severe environmental conditions in the wild population. One important exception is that very low values of *N*_eV_ are consistently observed in the wild population, suggesting that underlying causes of genetic decline continue to operate despite supplementation.

### Population density, genetic diversity, and effective size

Extensive demographic surveys show that the wild population of Rio Grande silvery minnow has experienced multiple changes in density that exceed an order of magnitude over the past two decades (U.S. Fish and Wildlife Service 2010). From 2000 to 2004, densities of Rio Grande silvery minnow were less than one fish per 100 m^2^, and during this time, the threat of extinction in the wild was acute. A key premise of genetic monitoring is that population declines will be accompanied by erosion of genetic diversity and reduction in genetic effective size. However, in Rio Grande silvery minnow, strong positive relationships between population density, genetic metrics, and effective size were not observed over the 12-year time frame of the study.

It is apparent for both microsatellites and mtDNA that there is considerable inter-annual variability in gene diversity metrics and effective size estimates from 1987 and 1999–2004 (Fig. 1). However, following the onset of population supplementation with captively reared fishes, the general trend was toward stabilization and marginal increases in mtDNA and microsatellite diversity and number of alleles/haplotypes. Inter-annual variability in all of these measures decreased after 2005.

Temporal method estimates of genetic effective size remained very low over the entire study but increased slightly in more recent samples. Three estimates of *N*_eV_ (derived from TempoFs) revealed an effective size of <100 (1999–2000, 2001–2002, 2002–2003), and five (TempoFs) and three (moments) comparisons were <200 during periods of low wild fish density (<1 to ∼6 fish per 100 m^2^) and no supplementation. From 2004 to 2010, estimates of *N*_eV_ remained low [mean, 180 (moments)-422 (mlne)] but less variable (range, 115–307) than in the 1987–2003 period (range, 368–1186). For more recent comparisons (2008–2010), estimates of *N*_eV_ from moments and TempoFs methods converged. Although mlne estimates were typically larger than those based on TempoFs or moments, they nevertheless indicate a decline in mean *N*_eV_ from (1987–2004 = 842) comparisons to recent estimates (2004–2010 = 422). One estimate based on TempoFs is larger (*N*_eV_ = 433) than other estimates obtained using this method and was recorded over a time frame (2003–2004) where some of the lowest densities of Rio Grande silvery minnow occurred. This result is somewhat counterintuitive, as this increase pre-dated a large boost in wild population densities in 2005 that resulted from favorable flow conditions in spring of that year and absence of river intermittency during the summer.

There are at least two reasons for the apparent disconnection of population density, genetic diversity, and effective size in Rio Grande silvery minnow. First, it may take several generations of very small population sizes for diversity to be depleted enough to be detected by genetic monitoring. It is well established that heterozygosity is a relatively insensitive indicator of population bottlenecks (Allendorf 1986), even ones of extremely small size. Secondly, it is an explicit goal of the supportive breeding program for Rio Grande silvery minnow to maintain genetic variability (USFWS 2009), so the lack of correlation between density and genetic diversity is likely due to supplementation practices and successful breeding of released fish. Supportive breeding may also explain lack of correlation of density and *N*_e_ estimates (discussed below).

### Population supplementation, diversity, and effective size

Supportive breeding has the potential to maintain diversity and to increase the effective population size either by increasing abundance or by reducing variance in reproductive success among parents (Ryman and Laikre 1991). In contrast, it may deplete genetic variation (Tessier et al. 1997) and depress the effective size (Ryman and Laikre 1991), depending on the genetic composition of fish repatriated from captivity. Ryman et al. (1995) found that risks to genetic diversity and effective size were greatest for species capable of producing large numbers of offspring in captivity (i.e., that exhibit type III survivorship); this characterizes the life history strategy of Rio Grande silvery minnow. We found that wild samples collected after the onset of population supplementation had significantly greater microsatellite diversity (*H*_ec_ and *N*_ac_) and mitochondrial diversity (*H*_R_) than those prior to supplementation, which supports the prediction that supplementation buffers the population against loss of genetic diversity following bottlenecks in Rio Grande silvery minnow. This may be because the supportive breeding program for Rio Grande silvery minnow differs in some respects from traditional hatchery programs that spawn a small portion of the wild population in captivity. In Rio Grande silvery minnow, the preferred source of fish for population supplementation are eggs collected from natural spawning events. These egg collections should represent reproductive effort of a large segment of the wild population and therefore have the potential to capture a representative sample of its genetic variability. For example in 2002, more than 900 000 eggs were collected from the wild, and 230 000 of them were repatriated to the Rio Grande between 2003 and 2004. Some were retained by conservation hatcheries in New Mexico for use as the founding captive broodstock. Subsequent wild-breeding of captive-released fish likely made a significant genetic contribution to the wild population, and collection of eggs prior (2001–2003) to the population collapse that occurred from 2002 to 2004 may have helped to preserve diversity that would otherwise have been lost during these severe population contractions. Similarly, maintenance of diversity through periods of population supplementation has been demonstrated in both Chinook and Chum salmon (Eldridge and Killebrew 2008; Small et al. 2009).

Supportive breeding aims to reduce early-life mortality and associated variance in reproductive success, or the ‘sweepstakes mismatch’ process (Hedgecock 1994), that characterizes reproduction in Rio Grande silvery minnow in its currently fragmented habitat (Osborne et al. 2005; Turner et al. 2006). Although type III survivorship and a consequent small *N*_e_:*N*_c_ ratio (Alò and Turner 2005) are typical for the species in the wild, supportive breeding likely ameliorated characteristically high variance in reproductive success by capturing a large portion of the species’ reproductive effort before it is transported to unsuitable habitat (Turner et al. 2006) and then rearing these progeny in protective custody. It is plausible that supplementation explains the observed increase in *N*_eV_ for 2003–2004 in the wild. Wild population densities were sufficiently low in 2003 such that repatriated fish probably comprised a large portion of the population. Adult fish released to the wild from captivity in 2003 and 2004 were derived from wild-caught eggs collected from 2001 to 2003. Temporal comparison of allele frequencies between 2003 and 2004 was therefore based largely on progeny of repatriated fish and not wild fish, and thus yielded larger estimates of *N*_eV_.

Between 2005 and 2010, estimates of *N*_eV_ were smaller than for the 2003–2004 comparison. During this time, densities of Rio Grande silvery minnow in the wild fluctuated greatly with densities ranging from a high of nearly 37 fish per 100 m^2^ (2005) to around one fish per 100 m^2^ (2006 and 2010). Over one million captive-spawned fish were released compared to only around 26 000 progeny of wild-caught and captive-raised eggs during the same time period. These fish were progeny of a captive broodstock derived from eggs collected in 2002. In years when recruitment of wild fish was poor (e.g., in 2006), progeny (produced in the wild) of captive-bred fish potentially comprised a disproportionate fraction of the subsequent generation. Despite efforts to maximize diversity of captive stocks, observed depression of *N*_e_ could reflect a Ryman–Laikre effect (Ryman and Laikre 1991), which occurs when variance in reproductive success of a population is increased owing to disproportionate contribution of offspring from relatively few captive breeders.

There are alternative hypotheses that could explain the apparent discrepancy between *N*_e_ and density in Rio Grande silvery minnow. For example, density-dependent effects such as genetic compensation can also cause *N*_e_ to be decoupled from *N*_c_ (Palstra et al. 2009; Saarinen et al. 2010). Compensation occurs when reduction in the effective number of breeders (*N*_b_) is counterbalanced by reduced competition for mates or spawning sites when *N*_c_ is small, reducing among-spawner differences in offspring survivorship. It is not possible to distinguish between these two effects (compensation versus supplementation) with the current data set. However, stabilization of genetic diversity metrics and *N*_eV_ coincides with the onset of supplementation and occurs despite dramatic fluctuations in the wild. Thus, supplementation appears to be the most plausible explanation for the results.

### Inter-relationships of effective size estimates

*N*_eV_ estimates obtained using different methods should be positively inter-correlated, but this was not observed for values obtained over the entire 12-year time series. However, after the commencement of supplementation, all three commonly used estimators of *N*_eV_ produced positively correlated values. Lack of correlation overall may be due to known biases of methods used to estimate *N*_eV_ (e.g., Waples 1989; Turner et al. 2001; Wang 2001; Jorde and Ryman 2007). Likewise, *N*_eV_ and *N*_eD_ estimates were not correlated for any period; this result is not unexpected, as these estimates use different aspects of the data to estimate variance and inbreeding effective size.

Estimators of *N*_eV_ used in this study are subject to specific biases that influence accuracy and precision in different ways. For example, mlne tends to overestimate *N*_e_ when calculated from loci with highly skewed allele frequencies (Jorde and Ryman 2007) and can provide imprecise estimates in nonequilibrium populations (Wang 2001). Both Waples (1989) and Turner et al. (2001) noted that moments estimates obtained using the most commonly employed measures of allele frequency change (Nei and Tajima 1981; Pollak 1983) tended to be downward biased (resulting in overestimates of *N*_e_) when allele frequencies are close to zero or one. Jorde and Ryman (2007) and Antao et al. (2010) also noted that unbiased estimates of effective size (TempoFs) may come at the cost of precision, with wider confidence intervals than moments estimates. Regardless, in all except two cases (2003, 2004) for which sufficient samples were available, noninfinite upper-bound CIs were obtained.

Several benchmarks for ascertaining extinction risk have been suggested for interpreting effective size in threatened species. The most conservative targets deemed necessary to maintain long-term genetic security of a species range from an effective size of 500–5000 (Franklin and Frankham 1998; Lynch and Lande 1998). Regardless of potential biases, all *N*_eV_ estimates suggest values of *N*_e_ that are smaller than the minimum benchmark of *N*_e_ = 500 in seven of eight (mlne) and all (moments) of the most recent temporal comparisons.

Values of *N*_eD_, which provide a measure of the inbreeding effective size, were uniformly higher than and not positively correlated with estimates of *N*_eV_. The underlying principle of the LD method is that as *N*_e_ decreases, genetic drift increases nonrandom association among alleles at different loci (Hill 1981). As erosion of linkage disequilibrium can take several generations, *N*_eD_ may also contain information on the effective size from several generations that precede a population decline. In addition to this upward bias, single-sample *N*_e_ estimators including *N*_eD_ provide an estimate of the effective number of parents that produced the progeny from which the sample is drawn (Waples 2005). Estimates of *N*_eV_ and *N*_eD_ were paired accordingly (refer to Methods for details) to account for this potential source of bias, so this is unlikely to explain the lack of correlation between *N*_eV_ and *N*_eD_ estimates.

Using computer simulations, Antao et al. (2010) evaluated the ability of *N*_eD_ and *N*_eV_ estimators to (i) detect a population decline, (ii) correctly identify a bottleneck with low bias and high precision, and (iii) evaluate whether the methods were subject to a high rate of false positives (i.e., indicate a bottleneck when none had occurred). They found that temporal-method estimates of *N*_eV_ were very close to the bottlenecked population size in the first generation, whereas *N*_eD_ always overestimated *N*_e_ with relatively low precision. Moreover, in the first generation following a decline, values of *N*_eD_ were much closer to the prebottleneck population size. This result is consistent with the idea that estimates of *N*_eD_ include information on the effective size of previous generations (Waples 2005).

From a management perspective, there are a number of theoretical and practical distinctions between *N*_eI_ (to which *N*_eD_ estimates are most closely associated) and *N*_eV_. These two measures should be similar in stable populations but show predictable differences in declining (or growing) populations. Waples (2002) demonstrated that in declining populations, *N*_eV_ is reduced more rapidly than *N*_eI_, and, as a consequence, *N*_eV_ will be smaller than *N*_eI_ until equilibrium is reached. Conversely, Waples (2002) found the opposite for increasing populations, as increasing population size rapidly attenuates the magnitude of genetic drift, but inbreeding effects persist longer because of inherent relatedness among individuals derived from a bottlenecked (or reduced) population. Thus, the observed discrepancy between *N*_eV_ and *N*_eI_ in Rio Grande silvery minnow is precisely the signature expected for a declining population. In fact, the only significant correlation we found between *N*_eV_ (mlne) and *N*_eD_ was negative (*r* = −0.69, *P* = 0.047). At present, Rio Grande silvery minnow are subject to source–sink dynamics. Specifically, captive stocks (source) contribute breeders to the wild (sink) each year, where reproductive success and recruitment are highly variable. Under these circumstances, we would expect discrepancy between *N*_eV_ and *N*_eD_. Such dynamics are also likely to occur in other endangered but highly fecund species, especially fishes.

Although supplementation could confound relationships between *N*_eV_ and *N*_eI_ in a declining population, theoretical and empirical treatments of this issue are too limited to provide firm guidance with respect to Rio Grande silvery minnow. For example, a theoretical evaluation by Ryman et al. (1995) was limited to cases of critically low census sizes (*N*_c_ < 50); therefore, it is unclear how effects on severely bottlenecked populations relate to supplemented populations in which thousands to millions of individuals potentially contribute to annual reproduction, which is the case for Rio Grande silvery minnow. However, Waples and Do (1994), based on empirical evaluation of supplemented Pacific salmon, found that the most important factor that determines effects on *N*_eI_ after supplementation is whether the population maintains a large size; this finding is consistent with observations of larger *N*_eI_ than *N*_eV_ in the hatchery supplemented Rio Grande silvery minnow. A simulation study is currently underway (by EWC, MJO and TFT) to determine relationships between *N*_eI_, *N*_eV_, and supplementation in this species.

The value of *N*_eD_ dropped substantially in 2004 (*N*_eD_ = 595) from estimates of ∼2000 in prior years. This coincided with very poor wild recruitment into the 2002 and 2003 year-classes. Subsequent increases in *N*_eD_ ranged from 2000 to 4400 and coincided with an increase in wild fish densities (2005), the input of large numbers of captive-spawned fish (∼10^6^ between 2005 and 2010), and somewhat more favorable environmental conditions (e.g., less extensive channel drying).

Estimates of *N*_e_ varied across methods, but all estimators indicated a genetic effective size that is one or more orders of magnitude smaller than the census size (estimated between ∼24 000 and 3.5 million, Dudley et al. 2011). In contrast to the *N*_eD_ estimates (which were generally in the 1000’s), all but three *N*_eV_ estimates (across all estimators) were lower than 500 and three of the unbiased estimates from TempoFs were <100. We can conclude, therefore, that the genetic effective size of the wild Rio Grande silvery minnow population is smaller than expected from census size. Relatively large values of *N*_eD_ reflect a population in decline rather than evidence of robust genetic resistance to extinction.

The results are consistent with our previous studies that have shown that *N*_eV_ in wild Rio Grande silvery minnow is up to three orders of magnitude lower than adult census size (Alò and Turner 2005; Turner et al. 2006; Osborne et al. 2005). To explain this result, we proposed a model whereby the vast majority of reproductive output from spatially discrete spawning aggregations, comprised of semi-buoyant eggs and larvae, move passively downstream, past dams to relatively poor nursery habitat (such as reservoirs) or areas with a higher propensity for channel drying (such as the San Acacia reach). Once displaced, progeny either fail to recruit or cannot migrate back upstream to the natal reach. The model predicts that negative effects of downstream transport of reproductive output on *N*_eV_ are largely density independent. In other words, loss of productivity and variance among spawning aggregates in the wild should persist despite enormous supplementation from captive sources. Low values of *N*_eV_ observed in the wild prior to and after the onset of supplementation support this idea. In the absence of supplementation, we would also expect substantial losses of genetic diversity and values of *N*_eD_ to converge with those obtained from *N*_eV_ estimators if this model is correct.

The downstream distance travelled by Rio Grande silvery minnow eggs and larvae is determined, in part, by development time required for hatching and transition from a yolk-sac larva to a free-swimming stage. Time required usually exceeds 4 days, and downstream drift distances can exceed 100 kms (Dudley and Platania 2007b). Passively drifting propagules are swept past diversion dams that occur roughly every 60–90 kms in the current range of the species. In other species of pelagophiles, and likely in Rio Grande silvery minnow prior to supplementation, diversion dams are highly likely to be responsible for population declines in upstream reaches because these structures prevent upstream movement of any spawned fish displaced over dams as eggs or larvae (Dudley and Platania 2007b). Hence, genetic diversity in subpopulations in upstream reaches should be eroded in the absence of inputs from the hatchery or downstream sources and will eventually impact the entire population if upstream subpopulations represent a source and the downstream subpopulations act as a sink. In the Rio Grande, there are also reach-specific environmental effects such that flow conditions are more reliable in the Angostura reach, but drying is more likely in the San Acacia reach. These dynamics have predictable effects on genetic diversity. For example, mtDNA diversity in Rio Grande silvery minnow is highly variable across the time series in the San Acacia reach, whilst there appears to be more stability in the Isleta reach. The Isleta reach is less subject (compared to the San Acacia reach) to severe drying events.

### Broodstock effective size and *N*_eD_

As predicted, a significant and positive correlation was observed between *N*_eD_ estimates and the number of broodstock used for matings in captive brood lots. Likewise, stocks reared from wild-caught eggs tended to have larger effective sizes than those produced through captive spawning. This is not surprising as wild-caught eggs should reflect a large number of wild parents. Interestingly, many *N*_eD_ estimates were less than the broodstock census size. A number of these instances involved paired matings, which suggests that the linkage disequilibrium method may underestimate the true effective size or alternatively, and perhaps more likely, that there is some variance in reproductive success (i.e., not all breeding pairs contribute equally) among captive spawners.

## Conclusions

The preponderance of evidence suggests that the trajectory of genetic change in Rio Grande silvery minnow was primarily determined by supplementation from captively reared stocks, and not by fluctuations of population density of wild fishes. Standing levels of genetic diversity (heterozygosity and allelic richness) observed prior to supplementation were maintained or increased slightly over the study. These results suggest that inbreeding genetic effective size is large enough to preclude significant losses of genetic diversity in the near term. However, variance effective size remained lower than inbreeding effective size and substantially lower than population density, suggesting that the interaction between early life history and river fragmentation is still exerting downward pressure on this metric, despite supplementation. In the absence of supplementation, we predict convergence of inbreeding and variance effective sizes and substantive losses of genetic diversity each generation thereafter.

In more general terms, however, some of the genetic signals of population decline (i.e., loss of diversity) in the wild may not be detectable using genetic monitoring when the population is being heavily supplemented from captive stocks. In order to fully assess the effects of population supplementation (or any other management action aimed at maintaining genetic diversity), it is necessary to assay baseline samples obtained prior to supplementation. Such samples are typically not available (but see Gow et al. 2011) because conservation hatcheries are often not implemented and genetic data not collected until a species has declined sufficiently to warrant management actions. Our results also bode poorly for use of genetic monitoring as a singular estimator of population density or size when multiple factors impinge on genetic characteristics of the population. In such cases, a combination of traditional population monitoring, careful record-keeping in hatchery facilities, and genetic monitoring is required to completely assess trajectories of metrics associated with extinction risk, maintenance, and/or recovery of a managed population.

## Data Availability

Data for this study are available on Dryad: doi: 10.5061/dryad.p57j80c4.

## Supporting Information

**Appendix S1.** Stocking history of Rio Grande silvery minnow.

**Table S1.** Augmentation of Rio Grande silvery minnow by river reach.

**Table S2.** Summary statistics for microsatellite loci for wild, hatchery reared wild-caught eggs (WcE), captively spawned (Cs) Rio Grande silvery minnow.

**Table S3.** *N*_eD_ estimates estimated using all loci and with Lco8 excluded and associated 95% confidence intervals.
