# Supplementary material for: Genetic monitoring and complex population dynamics: insights from a 12-year study of the Rio Grande silvery minnow
Source: Evol Appl. 2012 Jan 12;5(6):553–74. doi: 10.1111/j.1752-4571.2011.00235.x (PMC3461139; doi:10.1111/j.1752-4571.2011.00235.x)
Supplement: Supplementary file 1 [file eva0005-0553-SD1.doc]

**S1**

***Stocking History of Rio Grande silvery minnow***

Captive stocks of Rio Grande silvery minnow originated with eggs and wild adults collected in

May 2000. These fish were placed in propagation facilities to act as broodstock and to serve as a

refugial population. Between May and June 2000 eight groups of silvery minnow were

artificially induced to spawn (broodstock collected from the San Acacia reach, number of

individuals released, N = 522) (Platania & Dudley 2001). Larval fish from these efforts were

released at Bernalillo (N = 91,600) and Los Lunas (N = 112,000). In January 2002, 12,900 (reared from wild-caught eggs) Rio Grande silvery minnow were released in the San Acacia reach of the middle Rio Grande, New Mexico by the University of New Mexico and Museum of Southwestern Biology (this was not part of the official augmentation program). Experimental augmentation by U. S. Fish and Wildlife Service Fishery Resource Office began in June 2002 with release of 2,082 adult fish in the Angostura reach (Alameda Bridge) followed by further releases in December 2002 and January 2003 (N = 103,639) and in April 2003 (N = 2,266) (Davenport & Brooks 2003). In January and April 2004, a further 117,829 fish were released (U.S. FWS Dexter National Fish Hatchery and Technology Center Field Note 4 April 2004; 2004 Release schedule for Rio Grande silvery minnow provided to Controlled Propagation Working Group February 11 2004, Ecological Services Office by J. Remshardt U.S FWS NMFRO; U.S. FWS NMFRO trip report May 7 2004). Between 2000 and 2004 over 400,000 captively reared and/or spawned fish have been released in the middle Rio Grande. Marking allowed all hatchery-reared fish to be distinguished from wild individuals. Commencing in 2005, augmentation was expanded to encompass both the Isleta and San Acacia reaches. Between 2005 and 2007, 100,000 to 400,000 Rio Grande silvery minnow were released annually throughout all reaches (Remshardt 2008). In 2008, no fish were released to the middle Rio Grande.

Fish reared from wild-caught eggs comprised the majority of released fish from 2002-2004 (2002- ~12,900 fish, 2003- ~163,000 fish, 2004- ~90,000 fish). The year refers to the year the released fish will enter the spawning population (for example fish released in Dec. 2002 or Jan. 2003 will both enter the 2003 spawning population). Since 2004, the vast majority of fish released as part of the augmentation program have been derived from captive spawning. Wild-caught eggs and larval fish have been collected since this time but in relatively small numbers and have therefore been retained to serve as captive broodstock.

All fish released were marked with visible implant elastomer (VIE) tags (2002) or calcein (all fish released in 2004). A portion of calcein marked fish were also VIE tagged.

Further information on the captive breeding and augmentation program (including the Rio Grande silvery minnow genetics and captive management plan) can be found at <http://www.middleriogrande.com/>

**Table S1.** Augmentation of Rio Grande silvery minnow by river reach.

|  | Angostura | Isleta | San Acacia |
| --- | --- | --- | --- |
| 2002 | Y | - | - |
| 2003 | Y | Y | - |
| 2004 | Y | - | - |
| 2005 | Y | Y | Y |
| 2006 | Y | Y | Y |
| 2007 | Y | Y | Y |
| 2008 | - | Y | Y |
| 2009 | - | - | - |
| 2010 | - | Y | Y |

**Table S2.**  Summary statistics for microsatellite loci for wild, hatchery reared wild-caught eggs (WcE), captively spawned (Cs) Rio Grande silvery minnow. Sample size (*N*), expected heterozygosity (*H_E_*), observed heterozygosity (*H_O_*), allelic richness (*A_R_*) and average weighted inbreeding co-efficient (*F_IS_*) are given over all loci. *N_eD_* estimates (based on nine microsatellite loci) and associated 95% confidence intervals (obtained using jack-knifing) are given. *WcE-01 sample was also collected from San Acacia but reared at Dexter (WcE-SA-01 was reared at the Albuquerque Biopark). (An- Angostura, SA- San Acacia, numerals following refer to the years eggs were collected, for example WcE-SA-01 were wild-caught eggs collected from the San Acacia reach in 2001).

|  | Microsatellites | | | | | |  |
| --- | --- | --- | --- | --- | --- | --- | --- |
| **Population** | **N** | |  | ***H_E_*** | ***H_O_*** | ***A_R_*** |  |
|  |  | |  |  |  |  |  |
| **1987** | 43 | |  | 0.797 | 0.710 | - |  |
| **1999** | 46 | |  | 0.815 | 0.647 | - |  |
| **2000** | 194 | |  | 0.815 | 0.697 | 13.298 |  |
| **2001** | 128 | |  | 0.808 | 0.721 | 13.729 |  |
| **2002** | 389 | |  | 0.794 | 0.680 | 13.676 |  |
| **2003** | 169 | |  | 0.818 | 0.709 | 13.902 |  |
| **2004** | 162 | |  | 0.820 | 0.738 | 13.792 |  |
| **2005** | 394 | |  | 0.817 | 0.725 | 13.947 |  |
| **2006** | 383 | |  | 0.826 | 0.726 | 14.040 |  |
| **2007** | 218 | |  | 0.829 | 0.727 | 13.821 |  |
| **2008** | 474 | |  | 0.824 | 0.713 | 14.043 |  |
| **2009** | 476 | |  | 0.832 | 0.689 | 14.049 |  |
| **2010** | 440 | |  | 0.837 | 0.693 | 14.155 |  |
|  |  | |  |  |  |  |  |
| ***WILD-CAUGHT EGGS*** | | | |  |  |  |  |
|  |  | |  |  |  |  |  |
| WcE-01* | 178 | |  | 0.820 | 0.651 | 13.766 |  |
| WcE-SA-01 | 50 | |  | 0.831 | 0.727 | 13.038 |  |
| WcE-An-02 | 50 | |  | 0.784 | 0.730 | 11.065 |  |
| WcE-SA-02 | 81 | |  | 0.819 | 0.680 | 13.907 |  |
| WcE-SA-03 | 51 | |  | 0.830 | 0.696 | 13.868 |  |
| MJO-07-005 | 54 | |  | 0.827 | 0.739 | 13.801 |  |
| MJO-07-006 | 49 | |  | 0.814 | 0.723 | 14.171 |  |
|  |  | |  |  |  |  |  |
| ***CAPTIVE SPAWNED*** | | |  |  |  |  |  |
|  | |  |  |  |  |  |  |
| MJO-06-29 | | 50 |  | 0.804 | 0.745 | 10.394 |  |
| Cs-01 | | 64 |  | 0.794 | 0.659 | 11.931 |  |
| Cs-An-02 | | 51 |  | 0.686 | 0.675 | 7.507 |  |
| Cs-SA-02 | | 53 |  | 0.803 | 0.673 | 12.034 |  |
| TFT039 | | 51 |  | 0.806 | 0.700 | 11.691 |  |
| Cs-04 | | 50 |  | 0.824 | 0.691 | 13.247 |  |
| TFT-04-23 | | 50 |  | 0.779 | 0.683 | 11.071 |  |
| TFT-04-24 | | 48 |  | 0.828 | 0.717 | 11.087 |  |
| TFT-04-25 | | 50 |  | 0.810 | 0.768 | 10.661 |  |
| TFT-04-29 | | 54 |  | 0.839 | 0.763 | 13.028 |  |
| TFT-04-30 | | 56 |  | 0.826 | 0.727 | 13.524 |  |
| TFT-04-31 | | 50 |  | 0.805 | 0.701 | 11.998 |  |
| TFT-05-006 | | 50 |  | 0.792 | 0.649 | 9.768 |  |
| TFT-05-007 | | 49 |  | 0.797 | 0.705 | 11.305 |  |
| TFT-05-008 | | 50 |  | 0.804 | 0.663 | 10.584 |  |
| TFT-05-009 | | 50 |  | 0.804 | 0.717 | 11.899 |  |
| TFT-05-011 | | 51 |  | 0.808 | 0.693 | 11.447 |  |
| MJO-06-25 | | 50 |  | 0.814 | 0.721 | 13.282 |  |
| MJO-06-028 | | 50 |  | 0.805 | 0.705 | 11.295 |  |
| MJO-07-007 | | 50 |  | 0.813 | 0.739 | 11.993 |  |

**Table S3.** NeD estimates estimated using all loci and with Lco8 excluded and associated 95% confidence intervals.

| **Sample** | **NeD(without *Lco8)*** | | **Jack-knife** | | **NeD(with Lco8)** | | **Jack-knife** | |
| --- | --- | --- | --- | --- | --- | --- | --- | --- |
|  |  |  | **-95%** | **95%** |  |  | **-95%** | **95%** |
| **1987** | Infinite |  | 79.8 | Infinite |  | Infinite | 139.3 | Infinite |
| **1999** | Infinite |  | -67 | Infinite |  | Infinite | -71.3 | Infinite |
| **2000** | Infinite |  | 77720.8 | Infinite |  | Infinite | -3145.6 | Infinite |
| **2001** | 1556.7 |  | 416 | Infinite |  | 2007.7 | 495.1 | Infinite |
| **2002** | 1510.4 |  | 535.2 | Infinite |  | 1950.6 | 701.7 | Infinite |
| **2003** | 2237.8 |  | 449.7 | Infinite |  | 2997.7 | 563.8 | Infinite |
| **2004** | 540.5 |  | 318.5 | 1498.1 |  | 595.5 | 357.2 | 1558.7 |
| **2005** | 3928.9 |  | 1058.7 | Infinite |  | 2724.3 | 1013.5 | Infinite |
| **2006** | 3762.2 |  | 1401.3 | Infinite |  | 2561.7 | 1291.4 | 34063.9 |
| **2007** | Infinite |  | 1217.5 | Infinite |  | Infinite | 1210.7 | Infinite |
| **2008** | 2697 |  | 1033.7 | Infinite |  | 4434.1 | 1436.6 | Infinite |
| **2009** | 3004.8 |  | 1385.7 | Infinite |  | 3607.6 | 1676.9 | Infinite |
| **2010** | 5979.4 |  | 1102.4 | Infinite |  | Infinite | 4060.5 | Infinite |
| **WcE-01*** | 1030.3 |  | 540.7 | 6716.6 |  | 1379.6 | 655.6 | Infinite |
| **WcE-SA-01** | Infinite |  | 215.3 | Infinite |  | Infinite | 238.3 | Infinite |
| **WcE-An-02** | 69.1 |  | 44.1 | 133.9 |  | 85.6 | 54.1 | 173.4 |
| **WcE-SA-02** | Infinite |  | 355.9 | Infinite |  | -1808.7 | 461.7 | Infinite |
| **WcE-SA-03** | Infinite |  | 379.2 | Infinite |  | 5008.5 | 307.6 | Infinite |
| **MJO-07-005** | 1180.2 |  | 159 | Infinite |  | 1065 | 195.9 | Infinite |
| **MJO-07-006** | Infinite |  | 251.9 | Infinite |  | Infinite | 520.6 | Infinite |
| **MJO-06-29** | Infinite |  | 25.3 | 70.5 |  | 42.2 | 28.7 | 68.7 |
| **Cs-01** | 50.4 |  | 38.9 | 68.2 |  | 43.7 | 35.6 | 55 |
| **Cs-An-02** | 19.1 |  | 12.8 | 29.2 |  | 21.6 | 14.9 | 32.5 |
| **Cs-SA-02** | 59.6 |  | 45.3 | 82.9 |  | 72.7 | 52.5 | 110.9 |
| **TFT039** | 97.4 |  | 46.8 | 740.9 |  | 106.3 | 56 | 433.5 |
| **Cs-04** | 59.8 |  | 40 | 104 |  | 65.5 | 45.7 | 105.7 |
| **TFT-04-23** | 20.7 |  | 16.2 | 27.1 |  | 20.4 | 16.5 | 25.4 |
| **TFT-04-24** | 40 |  | 28.4 | 61.1 |  | 40.2 | 29.7 | 57.8 |
| **TFT-04-25** | 24.3 |  | 19.4 | 30.9 |  | 24.9 | 20 | 31.5 |
| **TFT-04-29** | Infinite |  | 3631.7 | Infinite |  | Infinite | 532.2 | Infinite |
| **TFT-04-30** | 163.2 |  | 104.4 | 341.3 |  | 323.1 | 134 | Infinite |
| **TFT-04-31** | 75.6 |  | 48 | 151.2 |  | 83.2 | 54.7 | 154.7 |
| **TFT-05-06** | 45.2 |  | 33.8 | 63.9 |  | 49.4 | 38.8 | 65.7 |
| **TFT-05-07** | 64.5 |  | 42.9 | 114.2 |  | 86.6 | 53.2 | 191.3 |
| **TFT-05-08** | 32 |  | 25.2 | 41.7 |  | 32.2 | 26.7 | 39.5 |
| **TFT-05-09** | 139.2 |  | 71.8 | 763.6 |  | 219.9 | 98.8 | Infinite |
| **TFT-05-11** | 182.1 |  | 89.4 | 2048.9 |  | 136.6 | 81 | 354 |
| **MJO-06-25** | 222.7 |  | 113.9 | 1644.3 |  | 184.5 | 110.1 | 487.9 |
| **MJO-06-28** | 83 |  | 52.1 | 170.2 |  | 87.6 | 57.2 | 164.3 |
| **MJO-07-07** | 56.3 |  | 46 | 70.9 |  | 60.4 | 48.3 | 78.5 |
